# Effect of diabetic duration on hemorheological properties and platelet aggregation in streptozotocin-induced diabetic rats

**DOI:** 10.1038/srep21913

**Published:** 2016-02-22

**Authors:** Eunseop Yeom, Hyeokjun Byeon, Sang Joon Lee

**Affiliations:** 1 Department of Mechanical Engineering, Pohang University of Science and Technology, Pohang, South Korea

## Abstract

Diabetes mellitus with abnormal glucose concentration is associated with changes in hemorheological properties, endothelial function, and platelets hyperactivity. Disturbances may significantly be responsible for diabetes-related vascular complications. In this study, hemorheological and hemodynamic properties were measured according to diabetic duration after streptozotocin treatment in rats. For *ex vivo* measurements, an extracorporeal model was adopted. Flow rate and blood viscosity were measured using a microfluidic device. Erythrocyte aggregation and morphological parameters of erythrocytes were measured by modified erythrocyte sedimentation rate and the phase-contrast holography under *in vitro* conditions. The platelet aggregation and mean pressure in the femoral artery were estimated under *ex vivo* conditions. Hemorheological properties including blood viscosity, erythrocyte aggregation and shape parameters for the control group are significantly different with those for diabetic groups. The changes with respect to diabetic duration were relatively unnoticeable. However, the platelet aggregation is strongly dependent on the diabetic duration. Based on these results, hyperglycemia exposure may induce hemorheological variations in early stages of diabetes mellitus. High platelet aggregation may become more pronounced according to the diabetic duration caused by variations in hemorheological properties resulting in endothelial dysfunction. This study would be helpful in understanding the effects of diabetic duration on biophysical properties.

Diabetes mellitus is characterized by disordered metabolism and high hyperglycemia resulting from either low insulin level or high insulin resistance. Diabetes mellitus is associated with abnormal endothelial function, increase of arterial stiffness, platelet hyper-reactivity and hemorheological changes^1^,^2^,^3^. These resultant disturbances may play a critical role in the etiology of diabetes-related vascular complications, including arteriosclerosis, cardiac autonomic neuropathy and myocardial infarction^4^,^5^.

Among the variations, the viscosity increase of both plasma and whole blood caused by marked changes in hemorheological parameters, such as hematocrit, plasma proteins, erythrocyte aggregation, and deformability, can lead to the development of microvascular complications^6^,^7^,^8^. Specifically, hemorheological changes contribute to the production of vasoactive materials, such as nitric oxide (NO), prostacyclin, and endothelin, by changing shear stress on the endothelial cells^9^. The relationship between blood viscosity and peripheral vascular resistance was reported to be mediated by NO production^10^.

In previous studies using diabetic models, it was demonstrated that hyperglycemia leads to hyperaggregation and low deformability of erythrocytes by changing hemoglobin and membrane proteins of erythrocyte, and serum proteins (fibrinogen and globulins)^3^,^11^. Such hemorheological changes are implicated in the progression of retinal failure in diabetic retinopathy and renal failure in diabetic nephropathy.

Endothelial dysfunction mainly results from imbalance between reduced bioavailability of NO and abundant formation of reactive oxygen species (ROS) in the vascular wall. This dysfunctional phenomenon is accelerated in diabetics^12^. Impaired endothelial functions are observed in the very early stages of diabetes mellitus and hyperglycemia^13^. Given that NO and prostacyclin are inhibitors of platelet aggregation and leukocyte activation, the reduced bioavailability of NO in diabetes may result in platelet activation^1^. These activated platelets play a critical role in the progression of atherosclerotic lesion formation^14^. Therefore, a systematic monitoring of hemorheological properties such as blood viscosity, platelet adhesion, erythrocyte aggregation, and erythrocyte shape would provide a grasp of the pathophysiological features about the diabetes-related vascular complications.

To investigate between hemorheological properties and diabetes mellitus, various assessment techniques was adopted including rotational viscometer, ultrasonic diagnostic, erythrocyte sedimentation rate (ESR), filtration, 3D topography, optical tweezer, and microfluidic devices^8^,^11^,^15^,^16^. However, rotational viscometer requires a large amount of blood samples for repetitive tests. Ultrasonic diagnostic needs calibration procedure with varying flow speed and hematocrits, due to dependency of these factors. The ESR results are influenced by the installation angle and surface condition of test tubes. Since most techniques used to measure erythrocyte deformability handle single cells, it is difficult to measure statistically-averaged biophysical properties for many cells. In addition, they usually measure hemorheological properties under *in vitro* conditions. External exposure of blood samples can modify the aggregability and deformability of erythrocytes^17^.

For the measurement of biophysical properties under *ex vivo* condition, a rat extracorporeal loop model, circulating blood through an external loop that directly connects artery and vein, was employed in this study^18^. A number of biophysical properties were measured with the lapse of time by inserting a microfluidic device into the rat extracorporeal model. Given that the risk of developing vascular complications related with diabetes mellitus is associated with the degree and duration of hyperglycemia, we examined the effects of duration of hyperglycemia on biophysical properties, including blood viscosity, flow rate, pressure, degree of erythrocyte aggregation, erythrocyte shape, and platelet adhesion using the rat extracorporeal model and other measurement techniques.

## Results

### Rat conditions

The toxic effect of streptozotocin (STZ) treatment destroys pancreatic beta cells and changes physiological conditions of rat model^19^. Table 1 summarizes the biophysical characteristics for control and diabetic groups according to diabetic duration. The weight is gradually decreased in compliance with the duration of diabetes. Glucose concentration in blood samples was measured using an Accu-Chek® sensor instrument with test strips (Roche Diagnostics, Mannheim, Germany). Blood glucose for the control group is significantly lower than that for diabetic groups, but significant statistical difference is not observed among diabetic groups. Hematocrits are almost similar among all groups. The measured hematocrit values are relatively higher, compared with normal physiological condition^20^. It may be related with the dehydration of the rat due to a long-operational time. The level of fibrinogen in the plasma was measured by fibrinogen ELISA kit (Abcam, Cambridge, MA) in accordance with the manufacturer’s instructions. Fibrinogen concentration for the diabetic groups is significantly higher than that for control group. The platelet counts were measured by a semiautomated haematology analyzer (Medonic CA 620). The platelet numbers for diabetic groups are slightly lower than that for control group.

### *Ex vivo* blood viscosity

Blood viscosity and flow rate were repeatedly measured at intervals of 10 min for a total of 30 min after establishing the rat extracorporeal model. In this condition, response time, which is dependent on the flow rate, is less than 20 s. The time for observing the hydrodynamic balancing state is about 2 min. For minimization of dilution or blood loss due to *ex vivo* measurement, phosphate buffered saline (PBS; pH 7.4, Bio Solution, Korea) is only delivered at specific measurement instants. A blood sample was collected after 30 min of extracorporeal circulation to measure other biophysical properties including blood glucose, hematocrit, viscosity curve, aggregation, and erythrocyte shape.

**Figure 1:**
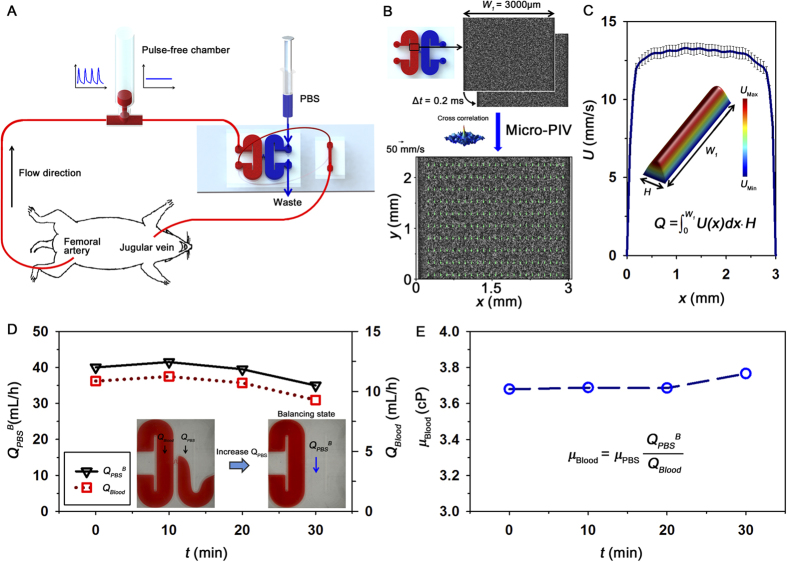
Experimental system for *ex vivo* monitoring of biophysical properties. (**A**) Blood is supplied into the extracorporeal fluidic network by connecting an extracorporeal loop to the blood vessels of a rat model. The extracorporeal loop consists of a pulse-free chamber (air cavity = 1.5 mL), and H-shaped and straight microchannels. The syringe and microfluidic devices were illustrated by the authors using SolidWorks software (Dassault Systèmes SolidWorks Corp., USA). (**B**) Procedure of micro-PIV measurement. The H-shaped microchannel has a width (*W*_*1*_) of 3000 μm and a height (*H*) of 80 μm. An instantaneous velocity field superimposed on the corresponding flow image is represented in the inset. (**C**) Axial velocity profile along the lateral position (*x*) of the channel. 3D velocity distribution in the channel and calculation of flow rate (*Q*). (**D**) Temporal variations in the flow rate of the PBS solution at the hydrodynamic balancing state (*Q*_*PBS*_^*B*^) and blood flow rate (*Q*_*Blood*_) measured by using Eq. (2). Microscopic images show hydrodynamic unbalancing and balancing states in the H-shaped microchannel. (**E**) Temporal variation of blood viscosity (μ_Blood_).

The viscosity of whole blood passing through the rat extracorporeal model was measured under *ex vivo* condition, as depicted in Fig. 1A. The flow rate of blood was estimated from the velocity profiles (Fig. 1B) measured using the micro-PIV technique. Figure 1C shows that the measured velocity profile in the center region of the microchannel is somewhat blunt because of the low aspect ratio (*H*/*W*_1_) of the rectangular channel. To measure blood viscosity, the hydrodynamic balancing state was induced in the H-shaped microfluidic device by adjusting the injection flow rate of PBS solution, as depicted in the bottom insets of Fig. 1D.

Figure 1D shows the flow rate of PBS solution at the hydrodynamic balancing state (*Q*_*PBS*_^*B*^) and the flow rate of blood (*Q*_*Blood*_) for the control rat sample. Although both flow rates *Q*_*PBS*_^*B*^ and *Q*_*Blood*_ slightly increase and decrease for 30 min, its variances are not very noticeable. The variation of flow rate may be caused by some external factors including anesthetization and environmental stress. By using Eq. (3), blood viscosity (*μ*_*Blood*_) can be estimated at specific measurement instants (Fig. 1E). Considering the slight variances of *Q*_*PBS*_^*B*^ and *Q*_*Blood*_, measured values of *μ*_*Blood*_ seem to be almost similar during the *ex vivo* measurements. The temporal variation of *μ*_*Blood*_ is inversely correlated with that of *Q*_*PBS*_^*B*^ and *Q*_*Blood*_, due to shear dependency of blood viscosity. Mean shear rate under *ex vivo* condition is approximately 2600 s^−1^.

### Variation of *in vitro* blood viscosity

**Figure 2:**
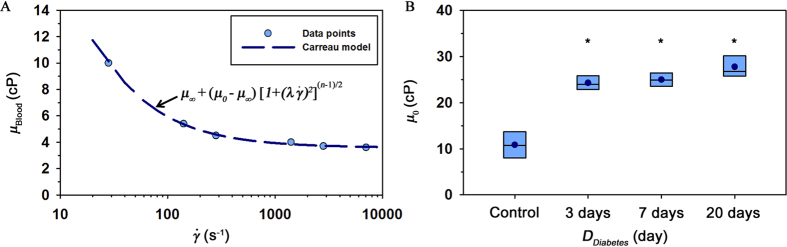
Variation of blood viscosity measured under *in vitro* condition. (**A**) Variation of blood viscosity according to shear rate. A curve fitted with Carreau model is included. **(B)** Viscosity values at zero shear rate (*μ*_*0*_) in the Carreau model are depicted according to duration of diabetes (*D*_*Diabetes*_). The line in the box represents median value. (*p < 0.001: significant difference from the control group).

Since the blood viscosity estimated under *ex vivo* condition depicts only the hemorheological property at a specific flow condition, the variation of blood viscosity according to shear rate was obtained by changing the flow rate of collected blood sample based on the relation between shear rate and flow rate. The input flow rate of a syringe pump was varied from 0.1 to 25 mL/h. Figure 2A shows variation of blood viscosity in a normal rat sample with respect to shear rate 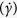. A number of different fitting equations have been used to depict blood viscosity according to shear rate^21^. Among these equations, the following Carreau model was adopted for blood viscosity prediction^22^.


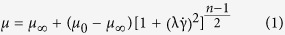


where, *μ*_*∞*_, *μ*_*0*_, *λ* and *n* represent viscosity values at infinite and zero shear rates, relaxation time, and power index, respectively. As expected, a line fitted with Carreau model is well matched with the measured viscosity data. A fitting parameter *μ*_*0*_ is depicted by the results in a box plot representation to compare different viscosity degree according to the duration of diabetes (*D*_Diabetes_) (Fig. 2B). Mean value of *μ*_*0*_for the control group is significantly lower than that for the diabetic groups. However, statistical difference between the diabetic groups is not observed.

### Variation of modified ESR

**Figure 3:**
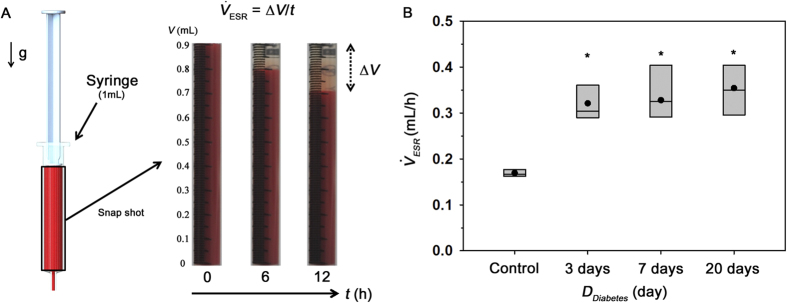
Measured modified ESR values. (**A**) Schematic of the modified ESR measurement. After loading a blood sample in a disposable syringe (1 mL), the syringe is vertically disposed in the inverted position. Typical images show the sedimentation of erythrocytes in the blood sample with respect to time (*t*). The syringe was illustrated by the authors using SolidWorks software. (**B)** Variation of ESR value 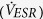 according to *D*_*Diabetes*_. (*p < 0.001: significant difference from the control group).

To determine the extent of erythrocyte aggregation, the modified ESR value was measured. Aggregated erythrocytes are easily sedimented by gravity (Fig. 3A). Right panel of Fig. 3A shows optical images with respect to time. As expected, the volume of the erythrocyte-depleted plasma (∆*V*) is gradually increased with lapse of time. Different degrees of erythrocyte aggregation between groups were compared by calculating 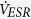 (Fig. 3C). Similar to the results of blood glucose and viscosity, 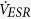 values for diabetic groups are markedly higher than that for the control group regardless of diabetic duration. The hyperaggregation of erythrocytes in diabetic groups can be elucidated by using the depletion theory. Since the osmotic force caused by the different concentrations of long-chain macromolecules between surface of erythrocyte and the bulk plasma makes adjacent erythrocytes aggregate together, the increased fibrinogen in STZ rat model gives rise to a high 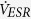
23,24.

### Variation of erythrocyte shapes

**Figure 4:**
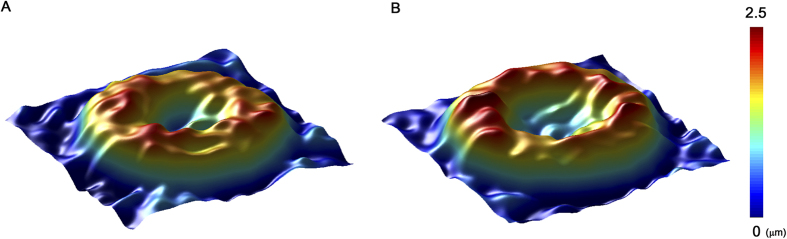
3D morphology of erythrocytes. A modified version of common-path diffraction phase microscopy was utilized to measure 3D morphology of individual erythrocytes. Overall magnification is approximately 120 × . Blood sample was diluted in PBS solution with volume ratio of 1:200. Topography of (**A**) a control and (**B**) diabetic erythrocyte after 20 days from STZ treatment. Color bar indicates the depth of the erythrocyte.

The cytoplasmic viscosity of erythrocyte is highly dependent on hyperglycaemia. Variation in the membrane lipid-protein interactions may alter viscoelastic properties of erythrocyte membrane. These abnormalities in erythrocyte membrane contribute to the changes in morphological features of erythrocytes in diabetic groups. The deformability of individual erythrocytes is closely related to the morphological feature of erythrocytes^11^. Specifically, the surface area-to-volume ratio of a biconcave disc significantly contributes to the degree of deformability^25^. In order to estimate the deformability of erythrocytes for each group, 3D shape of erythrocytes was measured by the phase-contrast digital holography. Figure 4 compares 3D morphologies of normal and diabetic erythrocytes. Since the shape changes according to the duration of diabetes are not significant, 3D morphology of a diabetic sample at the 20 days diabetic duration is only represented. Diabetic erythrocytes have relatively higher cell thickness compared to that of normal erythrocytes. However, significant shape changes, such as loss of biconcave shape and echinocyte shape transformation^26^, are not observed.

To more systematically investigate the morphological changes of erythrocytes, some morphological parameters, such as perimeter, 2D area, perimeter-to-2D area ratio, volume, 3D surface, and surface area-to-volume ratio, are summarized in Table 2. Perimeter, 2D area, volume, and 3D surface do not show noticeable changes in the consideration of their relatively high standard deviations. However, the perimeter-to-area ratio and surface area-to-volume ratio for the control group are significantly different with those for diabetic groups. Similar to other hemorheological properties, changes in morphological properties between diabetic groups are relatively weak with no statistical difference.

### Estimation of platelet aggregation under *ex vivo* condition

**Figure 5:**
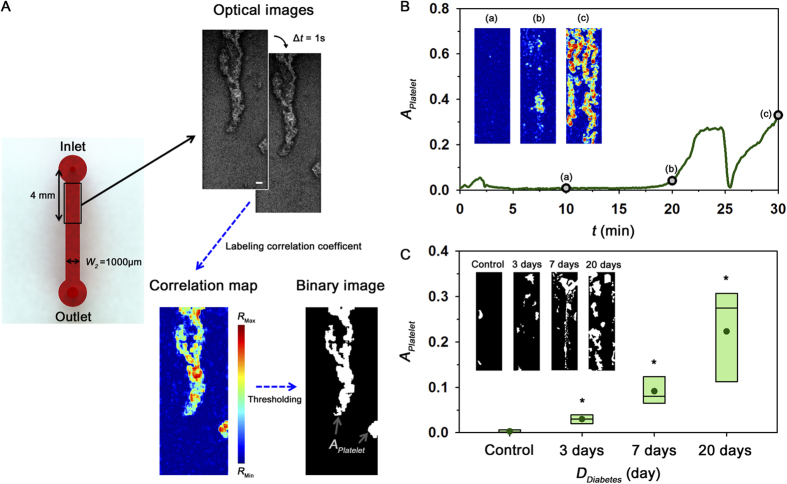
Quantification of platelet adhesion under *ex vivo* condition. (**A**) Procedure for measurement of platelet aggregation in the straight channel. Optical images were obtained by high-speed camera with time intervals (∆t) of 1 s. Scale bar indicates 100 μm. To distinguish adhered platelets from the flowing blood, a correlation map depicting the correlation coefficient of two consecutive images is obtained. By conducting additional image processing techniques, the area of adhered platelets (*A*_Platelet_) is obtained. (**B**) Temporal variation of *A*_Platelet_ for a rat model with 30 days diabetic duration. Correlation maps at 10, 20, 30 min are inserted. **(C)** Variation of *A*_Platelet_ according to the duration of diabetes *D*_*Diabetes*_. Typical binary images for the four groups are included. (*p < 0.005: significant difference from the control group).

As depicted in Fig. 5A, the degree of platelet aggregation was estimated by quantifying the area of adhered platelets in the straight channel under *ex vivo* condition. Temporal variations of the area of adhered platelets were monitored to find the optimal condition in advance of the main experiments. Figure 5B shows extremely severe variation in *A*_Platelet_ for a diabetic rat model with the 30 days diabetic duration. *A*_*Platelet*_ slightly increases at the initial state after establishing the rat extracorporeal model. Subsequently, the adhered platelets are almost not observed until 20 min later. As depicted in insets (Fig. 5B(a–c)), some platelets are rapidly attached in the straight channel after 20 min. Given that large platelet aggregates are subjected to large force caused by increased fluidic resistance, large aggregates are suddenly detached from the microchannel at around t = 25 min. However, platelets are re-adhered and then the straight channel is completely blocked by platelet aggregates at 40 min. Based on this temporal variation of *A*_Platelet_, the area of platelets *A*_Platelet_ at 30 min is used as the representative parameter to compare the platelet aggregation between the normal and diabetic groups.

Figure 5C shows a box plot representing *A*_Platelet_ at t = 30 min after the *ex vivo* measurement according to the duration of diabetes (*D*_*Diabetes*_). Unlike other hemorheological properties such as blood viscosity, erythrocyte aggregation, and erythrocyte shape, the degree of platelet adhesion for diabetic groups is largely increased with diabetic duration *D*_*Diabetes*_. As *D*_*Diabetes*_ increases, the standard deviation of *A*_Platelet_ is also significantly increased. Platelet activated by hemodynamic features is primarily mediated by soluble fibrinogen and von Willebrand factor (vWF) which support both adhesion and aggregation of platelets. Thus, the enhanced fibrinogen and vWF in diabetic rat models induce hyper-adhesion of platelets (Table 1)^27^.

### Observation of *ex vivo* hemodynamic conditions

**Figure 6:**
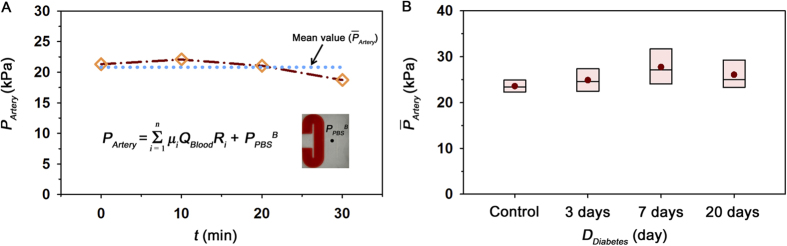
Estimation of mean pressure in the femoral artery under *ex vivo* condition. (**A**) Temporal variation of mean pressure at the femoral artery (*P*_*Artery*_) of the control rat sample, estimated based on the pressure drop in the fluidic circuit. The mean pressure 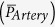 during the measurement is included. (**B**) Variation of 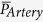 according to duration of diabetes *D*_*Diabetes*_.

Mean pressure variation was monitored during the *ex vivo* measurements. The different size of channels in the extracorporeal loop induces different shear rate at a certain part of the extracorporeal loop despite of the same flow rate. For reasonable estimation of mean pressure in the femoral artery (*P*_Artery_), the viscosity value at a specific shear condition fitted by Eq (1) was applied to Eq (5). Figure 6A shows temporal variation of *P*_Artery_ with the lapse of time under *ex vivo* condition for the control sample. Like flow rate results (Fig. 1D), *P*_Artery_ is slightly increased and then decreased in a 30 min period. A dotted line indicates the mean value of *P*_*Artery*_ 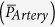 during the experiment time. 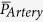 was used as a parameter representing the hemodynamic condition for all groups. As depicted in the box plot of Fig. 6B, statistically different tendency of 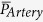 is not observed for all groups.

## Discussion

Considering that glucose is an essential nutrient for body cells, abnormal glucose concentration may have a direct influence on the constituents and biochemical properties of blood circulating in vascular networks^28^,^29^. In previous studies, it was found that STZ-induced diabetes mellitus significantly alter lipid composition, mRNA and plasma protein levels^16^,^30^,^31^. Specifically, increase in soluble thrombomodulin (sTM), vWF and fibrinogen and decrease in activated factor XIII have also been reported in STZ diabetic models^27^. These alterations can induce change in hemorheological properties such as viscosity, hematocrit, plasma proteins, erythrocyte aggregation, and deformability, and platelet aggregation^32^.

To investigate variation of hemorheological properties and platelet aggregation according diabetic duration, some measurement methods were adopted in the present study. Although *ex vivo* measurement can offer more reasonable data compared with *in vitro* measurement based on our previous studies^17^, it is difficult to simultaneously measure various hemorheological properties under *ex vivo* conditions. Therefore, viscosity variation, erythrocyte aggregation, and shape were measured by *in vitro* experiments. Fortunately, platelet adhesion can be quantified without significant flow modification when blood flow is similar among *ex vivo* measurements. To check flow conditions of *ex vivo* measurements, pressures at the femoral artery were compared.

Among these hemorheological properties, blood viscosity has a profound impact on blood circulation. In this regard, the variation of viscosity according to shear rate was measured using the H-shaped microchannel. Diabetic rats were found to have relatively high viscosities compared to the control. These results are in accordance with previous studies conducted on STZ-induced rats^16^. The increase in blood viscosity is consistently observed after 3 days from the STZ treatment. The elevated blood viscosity may be associated with the aggregability and deformability of erythrocytes^3^,^29^.

The ESR has been widely used as a nonspecific indicator of inflammation, because it is mainly affected by erythrocyte aggregation. Specific plasma proteins of fibrinogen and globulins form intercellular layer in the vicinity of erythrocytes, leading to an aggregating force. Therefore, the increase of these proteins contributes to enhanced erythrocyte aggregation in STZ diabetic models^33^,^34^. The contribution of the erythrocyte aggregation to the increase of the blood viscosity was evaluated by measuring the modified ESR. As expected, the modified ESR result exhibits similar variation trends of blood viscosity results according to diabetic duration.

In addition, diabetic state can cause reduced deformability of erythrocytes^35^,^36^. The deformability of erythrocytes is influenced by three factors: (i) the viscosity of intracellular fluid determined by the presence of hemoglobin, (ii) the viscoelastic properties of cell membrane, and (iii) the morphological feature of erythrocytes such as diameter, concave depth, surface area, and volume. In this study, morphological parameters were intensively investigated using phase-contrast digital holography. The erythrocytes suffered from hyperglycemia have different morphology compared to healthy erythrocytes^35^. It was reported that the perimeter-to-2D area ratio for erythrocytes becomes high with relatively low deformability, as the glucose concentration increases^37^. Similar to previous studies, the perimeter-to-2D area ratios for diabetic groups have smaller values than those for the normal group with high surface area-to-volume ratios. This morphological change in diabetic groups may be resulted from lipid alterations of erythrocyte membrane caused by decreased Na^+^-K^+^-ATPase activity^36^,^38^. However, significant variations are not observed among the diabetic groups with different diabetic duration. It signifies that the slightly reduced deformability of erythrocytes caused by the morphological shape change is continuously maintained after 3 days from the STZ treatment.

A sudden shear acceleration can directly initiate platelet aggregation, independent of biochemical triggers^39^,^40^,^41^. Therefore, platelets can be activated when they pass through the narrow inlet of the straight channel and adhered on the downstream of straight channel. By applying the correlation mapping, platelet aggregation can be estimated by measuring the area of adhered platelets. As shown in Fig. 5C, the degree of platelet adhesion and diabetic duration have a positive correlation (R^2^ = 0.623). Considering the process of platelet aggregation is dependent on the shear condition^42^,^43^, the mean pressure in the femoral artery was also estimated under *ex vivo* conditions to check the shear condition during the measurements of platelet adhesion. The measured mean pressures indicate that no significant difference exists among all groups tested in this study. Therefore, shear conditions may not have any significant influence on the increase trend of platelet aggregation according to the duration of diabetes.

Exposure to hyperglycemia can damage or alter endothelial barrier^12^. Increases in the blood viscosity and erythrocyte aggregation, as well as morphological shape change induced by diabetes mellitus, may also lead to endothelial dysfunction by disturbing the blood circulation in microvessels. Impaired endothelial dysfunction under diabetic conditions can result in reduced NO bioavailability and abundant ROS formation^13^. This type of imbalance between NO and ROS can lead to platelet aggregation with shape change of platelets, degranulation, and rapid surface expression of adhesion molecules^44^. In addition, the increases of vWF and fibrinogen due to endothelial dysfunction was observed in STZ diabetic models (Table 1)^27^. These proteins support both adhesion and aggregation of platelets. Thus, the hyper-adhesion of platelets may become more pronounced according to the diabetic duration caused by consistent exposure to hyperglycemia and large variations in hemorheological properties resulting in endothelial dysfunction. However, the results of platelet accumulation might be influenced by some artifacts caused by extracorporeal circulation of blood. Some portions of the platelet aggregates circulating in the rat model and extracorporeal loop can promote the aggregation of platelets. Thus, the measurement time under *ex vivo* condition is important to quantify the degree of platelet aggregation. Through preliminary experimental results, we found that the usage of a rat sample with high diabetic duration (more than 30 days) or prolonged experiment (more than 30 min) easily blocked the straight channel due to significant adhesion of platelet aggregates. Based on these results, a suitable experimental condition for estimating the platelet aggregation and blood pressure at the artery was determined.

## Conclusion

In this study, various biophysical properties, such as blood viscosity, erythrocyte aggregation, 3D morphology, and platelet aggregation, were measured according to the duration of diabetes under *ex vivo* and *in vitro* conditions. Based on these results, hyperglycemia induces variations in hemorheological properties from the very early stage of diabetes mellitus. It is caused by alteration of plasma proteins such as sTM, vWF and fibrinogen. These variations may lead to the hyper-adhesion of platelets and may be responsible for the diabetic-related vascular complications, such as nephropathy, retinopathy, and atherosclerosis in coronary and carotid arteries. Although further detailed study is required to examine the relationship between the variations of hemorheological properties and vascular complications of diabetic patients, we believe that this study would be beneficial in understanding temporal variations of hemorheological properties according to the duration of diabetes.

## Methods

### Fabrication of microfluidic devices

A rectangular master replica mold (height = 80 μm) was fabricated using microelectromechanical system (MEMS) technologies based on soft lithography and deep reactive-ion etching. An H-shaped microfluidic device has two identical side channels with width (*W*_1_) of 3000 μm and length of 14.4 mm. A bridge channel connecting both side channels has width of 100 μm and length of 2.4 mm. A straight microchannel has width (*W*_2_) of 1 mm and length of 10 mm. To initiate platelet aggregation in the straight microchannel, inlet region has narrow width about 100 μm and the microchannel was coated by collagen based on previous studies (Supplementary Fig. 1)^40^,^45^. After pouring Polydimethylsiloxane (PDMS; Sylgard 184, Dow Corning, USA) on the silicon replica mold, it was cured at 80 °C for 3 h. Thereafter, a PDMS block was peeled off from the silicon mold. The inlet and outlet of microchips were made with a puncher of 1.2 mm in diameter. After oxygen-plasma treatment (CUTE, Femto Science, Korea), the microfluidic device was finally prepared by bonding the PDMS block to a glass substrate.

Microfluidic devices employed for simultaneous measurements of biophysical properties, including blood pressure, viscosity, flow rate, and platelet adhesion have several distinctive advantages. First, biophysical properties of real blood can be directly measured by connecting the microfluidic devices with blood vessels of a rat model without noticeable hemorheological changes. The microfluidic devices can measure biophysical properties using small amount of sample consumptions. In addition, they do not require any calibration procedures.

### Preparation of extracorporeal model using diabetic rat samples

Male Sprague-Dawley rats were exposed to a 12/12 reverse-light cycle. Type 1 diabetes with hyperglycemia was induced by intraperitoneal injection of streptozotocin (STZ; 65 mg/kg in sterile saline) to rat samples under anesthesia with isoflurane and oxygen. Rat samples were fasted for 24 h prior to the STZ injection, and then fasted for another 24 h. They were divided into four groups, depending on the duration of diabetes (control, 3, 7 and 20 days).

A rat extracorporeal model was adopted to measure some biophysical properties under *ex vivo* conditions (Fig. 1A)^18^. All experiments were conducted using 16-week-old rat samples, which were anesthetized with intramuscular injection of ketamine (100 mg/kg) and xylazine (10 mg/kg). A PE-50 tube (ID = 0.58 mm, polyethylene tube) at one end of the extracorporeal loop was cannulated into the right jugular vein. Exact amount of heparin (1500 IU/mL/kg) was precisely injected into the right jugular vein for anticoagulation. After 10 min of heparin injection, another PE-50 tube at the other end of the loop was inserted into the left femoral artery. A pulsed-free microfluidic device with 1.5 mL air cavity was installed between the femoral artery and the inlet of the H-shaped microfluidic device to supply stabilized blood samples. The outlet of the H-shaped microchannel and the inlet of the straight microchannel were connected by a Tygon tube (ID = 250 μm). The blood passing through the extracorporeal conduits was returned to the jugular vein of the rat model. The cooling effect caused by the blood circulation through the extracorporeal loop was minimized by installing a heat chamber maintained at 36 °C to 37 °C. All experimental procedures were approved by the Animal Care and Ethics Committee of Pohang University of Science and Technology, and all methods were performed in accordance with the approved guidelines.

### Micro-PIV technique for flow rate measurement

As shown in Fig. 1B, a micro-PIV was used to measure blood flows in the H-shaped microchannel. The microfluidic devices were mounted on the stage of an optical microscope (Nikon, Tokyo, Japan) with 4 × objective lens (NA = 0.1). Optical images of blood flows were consecutively acquired using a high-speed camera (FASTCAM SA 1.1, Photron Ltd., San Diego, USA) at a frame rate of 5000 fps. Before applying the cross-correlation PIV algorithm to each image pair, the captured images were cropped into images of 408 pixels × 320 pixels. The detailed procedures of micro-PIV technique employed in the present study were well described in our previous studies^46^,^47^. The size of each interrogation window was 16 pixels × 32 pixels with 50% overlapping. The obtained velocity fields were filtered using a 3 × 3 median kernel. Velocity profiles were obtained by averaging the velocity field along the flow direction (y-axis).

Based on our previous study^15^, the measured velocity information can be postulated to represent the mean velocity of erythrocytes in the whole depth, because the depth of correlation (*δ*_C_ = 310 μm), which expresses the depth over which particles contribute to the cross-correlation analysis, is larger than the channel depth under present experiment condition. Therefore, the flow rate of blood (*Q*_*Blood*_) in the microchannel can be estimated using the following equation:


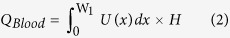


where *U*(*x*) is the velocity profile at a specific lateral position (*x*); and *W*_*1*_ and *H* indicate the width and depth of the H-shaped microchannel, respectively.

### Viscosity estimation

Blood sample and PBS solution were separately delivered into the two inlets of the H-shaped microfluidic device to estimate blood viscosity. The PBS solution was supplied using a syringe pump (neMESYS, Centoni Gmbh, Germany) with a 5 mL plastic syringe (BD). When a blood sample does not move in the bridge channel (hydrodynamic balancing state), the viscosity of the blood sample (*μ*_Blood_) can be simply estimated based on the following analytical formula:


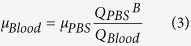


where, *μ*_PBS_, and *Q*_PBS_^B^ denote viscosity, and the flow rate of PBS solution at the hydrodynamic balancing condition, respectively. The viscosity of PBS solution (*μ*_PBS_) is approximately 1.00 ± 0.05 cP^48^. Details of viscosity estimation procedure were well described in our previous study^46^.

### Modified erythrocyte sedimentation rate measurement

Modified ESR value was measured using a 1 mL plastic syringe (BD). Based on our previous results, the hematocrit of the blood samples was adjusted to 20% for effective measurement of erythrocyte aggregation using the modified ESR value^15^. After loading blood sample (0.9 mL) into a disposable syringe, the syringe was disposed in an inverted vertical posture (Fig. 3A). Snap shots of the syringe were continuously captured with a digital camera (D700, Nikon, Japan) at 5 min intervals for 12 h. Boundaries between erythrocytes and erythrocyte-depleted plasma were determined by adopting Canny’s method^49^. ESR value 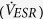 can be calculated by dividing the volume of erythrocyte-depleted plasma (*∆V*) by the measurement time (*t* = 12 h).

### Phase-contrast digital holography

Whole blood was diluted 1:200 in the PBS solution and then placed between slide glass and cover glass. Prepared blood sample was mounted on the stage of an upright microscope (Eclipse i50, Nikon) with 40 × objective lens (NA = 0.75). Additional relay optics were used to provide overall magnification of 120 × . A modified version of common-path diffraction-related phase microscopy was utilized to measure the 3D morphological shape of erythrocytes^50^. The laser beam (He–Ne laser; λ = 633 nm) is separated into two beams by a beam splitter. One beam passes through the test sample and the other beam, which is used as the reference beam, passes through a pinhole. A hologram, made by interference between the two beams, was captured by a charge-coupled device camera (PCO 2000, 2K × 2K pixel, 7.4 μm/pixel). An angular spectrum algorithm was employed for numerical reconstruction of hologram images. Perimeter, 2D projected area, 3D surface area and volume of erythrocytes were evaluated from the reconstructed phase images. The detailed procedures of hologram reconstruction and cell analysis were well described in our previous study^51^.

### Quantification of adhered platelets

Before establishing the rat extracorporeal model, the conduits, except for the straight microchannel, were incubated with 2% bovine serum albumin (Sigma, MO) for 1 h at room temperature to inhibit platelet deposition and fibrin adhesion. After 1 h, BSA is washed by PBS solution. A total of 60 images of blood flow and adhered platelets in the straight microchannel were consecutively acquired by the high-speed CMOS camera (FASTCAM SA 1.1) with time interval of 1 s at 30 min after establishing the extracorporeal rat model. In order to accurately distinguish the adhered platelets from the flowing blood, a correlation map, labeling the 2D correlation coefficient between small tiles of two consecutive images, was adopted (Fig. 5A)^52^. Each optical image was divided into small tiles of *m* × *n*. The 2D correlation coefficient (*R*) of tiles was calculated using the following equation:


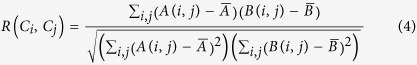


where *A* and *B* represent the tiles centered at (*C*_*i*_, *C*_*j*_) in two consecutive images, (*i*, *j*) is the pixel coordinate of the tile, and 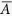 and 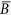 denote the mean intensity of the tiles *A* and *B*, respectively. The size of each tile used in this study is 11 pixels × 11 pixels. The correlation maps were converted into binary images by applying the thresholding with an optimal value of Otsu’s algorithm. Finally, area for adhered platelets (*A*_*Platelet*_) is estimated by calculating the total number of any nonzero pixels in the binary image.

### Estimation of mean pressure in the femoral artery

A discrete fluidic circuit of the present extracorporeal model is composed of fluidic resistances, air compliance of the pulse-free chamber, and flow rates. By adding pressure drops in the fluidic circuits from hydrodynamic balancing pressure in the microfluidic device, the mean pressure in the femoral artery (*P*_*Artery*_) can be estimated using the following equation;


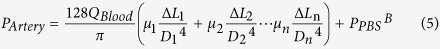


where, *μ*_n_*, L*_n_ and *D*_n_ represent the blood viscosity, characteristic length and hydraulics diameter at a certain part of the extracorporeal network (Supplementary Fig. 2). *P*_*PBS*_^*B*^ denotes the hydrodynamic balancing pressure in the H-shaped microchannel. In the prediction of blood pressure, it is assumed that blood is circulated in the extracorporeal network without bleeding and no change in the fluidic resistance. However, due to accumulation of platelet aggregates in the straight channel, the pressure in the artery (*P*_*Artery*_) would be somewhat overestimated.

### Statistical analysis

Results in each group indicate the mean value evaluated from five test samples. The statistical analysis of the data was carried out by Student t-test between control and diabetic groups. The statistical analysis among diabetic groups are not observed in all **Tables** and **Figures**.

## Additional Information

**How to cite this article**: Yeom, E. *et al.* Effect of diabetic duration on hemorheological properties and platelet aggregation in streptozotocin-induced diabetic rats. *Sci. Rep.* **6**, 21913; doi: 10.1038/srep21913 (2016).

## Supplementary Material

Supplementary Information

## Figures and Tables

**Table 1:** Biophysical characteristics of normal and diabetic rat groups.

	Control	DM (3 day)	DM (7 day)	DM (20 day)
Weight (g)	404.2 ± 15.7	364.9 ± 24.0	319.3 ± 38.1	241.9 ± 20.4^*^
Blood sugar (mg/dL)	67.7 ± 9.3	468.0 ± 49.2^*^	455.0 ± 41.4^*^	534.8 ± 39.4^*^
Hematocrit (–)	0.52 ± 0.01	0.52 ± 0.03	0.52 ± 0.04	0.49 ± 0.02
Fibrinogen (mg/mL)	3.24 ± 0.40	4.94 ± 0.25^*^	5.10 ± 0.25^*^	5.38 ± 0.30^*^
Platelets (×1000/μL)	735 ± 33	689.6 ± 54	704 ± 57	644 ± 49

Each value represents mean ± standard deviation.

^*^p < 0.001: significant difference from the control group.

**Table 2:** Morphological parameters of erythrocyte between normal and diabetic groups.

	Control	DM (3 days)	DM (7 days)	DM (20 days)
Perimeter (μm)	20.96 ± 1.11	20.42 ± 0.76	21.33 ± 0.81	20.73 ± 0.75
2D area (μm^2^)	44.01 ± 2.40	41.14 ± 3.25^**^	44.53 ± 2.07	42.38 ± 2.59
Perimeter/Area (μm^−1^)	0.48 ± 0.02	0.50 ± 0.02^**^	0.49 ± 0.02^*^	0.49 ± 0.01^**^
Volume (fL)	81.71 ± 12.59	88.88 ± 10.94	88.57 ± 8.26	87.03 ± 9.89
3D surface area (μm^2^)	111.20 ± 12.34	107.08 ± 9.07	111.32 ± 8.18	111.20 ± 5.91
Surface area/volume (μm^−1^)	1.37 ± 0.10	1.22 ± 0.16^**^	1.26 ± 0.12^**^	1.29 ± 0.15^*^

Each value represents mean ± standard deviation.

^*^p < 0.1, ^**^p < 0.05: significant difference from the control group.
