# Supplementary material for: Effect of diabetic duration on hemorheological properties and platelet aggregation in streptozotocin-induced diabetic rats
Source: Sci Rep. 2016 Feb 22;6:21913. doi: 10.1038/srep21913 (PMC4762006; doi:10.1038/srep21913)
Supplement: Supplementary Information [file srep21913-s1.pdf]

Effect of diabetic duration on hemorheological properties and platelet aggregation in streptozotocin-induced diabetic rats

Eunseop Yeom[1], Hyeokjun Byeon[1] and Sang Joon Lee[1]*

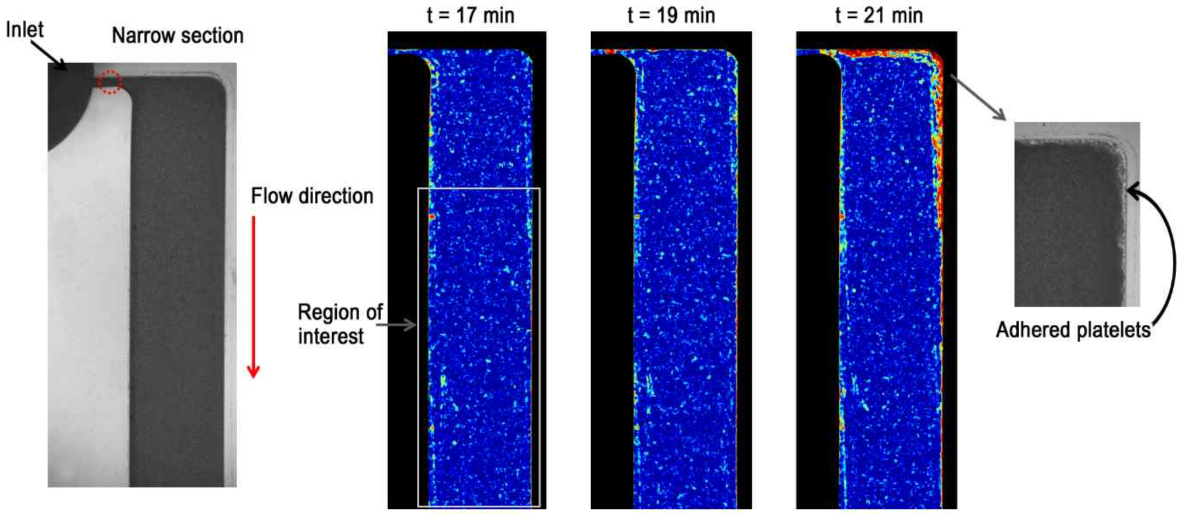

Supplementary Fig. 1 Temporal evolution of platelets adhesion in a rat model with diabetic duration of 20 days. The inlet of the straight channel has narrow section of 100 μm in width. Correlation maps at specific instants (t=17,19, and 21 min) and magnified optical image.

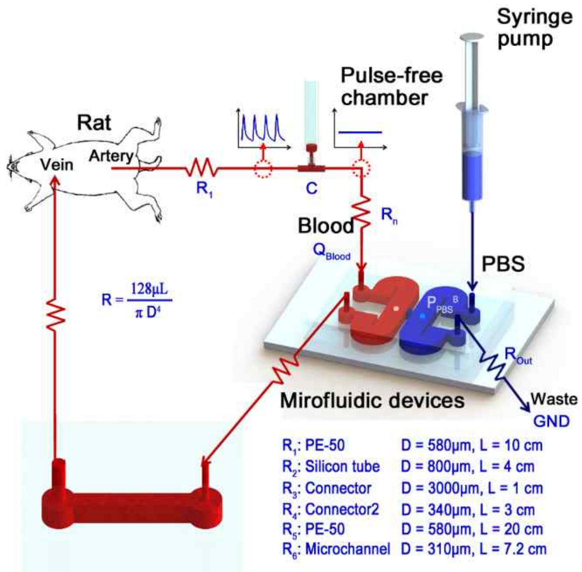

Supplementary Fig. 2 Discrete fluidic circuit of the proposed measurement system include fluidic resistances ($R_1 \cdots R_n$), air compliance (C), flow rate (Q_{Blood}), and the pressure at the hemodynamic balancing state (P_{PBS}^B). The syringe and microfluidic devices were created by the authors using SolidWorks software.
